# Awareness of Periodontal Health among Pregnant Females in Government Setting in United Arab Emirates

**DOI:** 10.1055/s-0043-1771451

**Published:** 2023-08-17

**Authors:** Sireen Al Raeesi, Khawla Al Matrooshi, Amar Hassan Khamis, Abdel Rahman Tawfik, Crawford Bain, Mohamed Jamal, Momen Atieh, Maanas Shah

**Affiliations:** 1 Emirates Heath Services, Ministry of Health and Prevention, Dubai, United Arab Emirates; 2 Hamdan Bin Mohammed College of Dental Medicine Dubai Healthcare City, Mohammed Bin Rashid University of Medicine and Health Sciences, Dubai, United Arab Emirates

**Keywords:** awareness, knowledge, pregnancy, periodontal disease, antenatal clinics

## Abstract

**Objective** Periodontal disease is one of the most common infectious diseases. Several factors are associated with increased susceptibility of periodontal disease such as hormonal changes during pregnancy. Although pregnancy does not directly cause gingivitis, it can aggravate preexisting periodontal disease. This study aimed to evaluate knowledge of the association between periodontal disease and pregnancy in pregnant females.

**Materials and Methods** A convenience sample of pregnant females attending two United Arab Emirates government hospitals was recruited for this study. A 23-item questionnaire was developed with four sections, covering sociodemographic details, oral hygiene, oral symptoms during pregnancy, and knowledge of periodontal health during pregnancy. The study was conducted between April and October 2017. All participants consented to the survey.

**Results** A total of 100 participants with a mean age of 31 years (± 5.9) completed the survey. Most respondents brushed their teeth 2 to 3 times a day (65%), used a manual toothbrush (93%) but only visited the dentist when in pain (62%). Few respondents self-reported any gingival signs and symptoms during pregnancy; 38% had bleeding gums, 27% had no gum swelling, and 34% had bad odor/taste/smell. Only 21% of pregnant females lost a tooth/teeth during pregnancy, 15% believed that pregnancy increased the likelihood of gum disease, and 66% of gynecologists did not advise a visit to the dentist.

Housewives were significantly less knowledgeable about periodontal health than students/employed respondents ( *p* = 0.01). Quality of knowledge was not associated with educational attainment (< 0.06). Respondents > 30 years of age were more likely to believe in “a tooth for a baby” than younger participants aged < 30 years ( *p* < 0.05). A logistic regression model showed that educational attainment was not a predictor for the belief in “a tooth for a baby” but age was a significant predictor (odds ratio = 2.0).

**Conclusion** Protocols should be developed in antenatal clinics in order to improve periodontal health in pregnant females and to prevent complications that can result in adverse pregnancy outcomes.

## Introduction

Periodontal diseases are a group of conditions that involve inflammation and destruction of the supporting structures of the teeth. These are considered multifactorial diseases, characterized by significant involvement of specific microbiota, environmental, and host factors. Research findings have demonstrated adequate evidence to suggest gingivitis and periodontitis not only lead to localized destructive patterns in the oral cavity, but also have systemic effects and are subsequently related to systemic manifestation of periodontal disease and conditions. 1 An amplified gingival response to biofilm-induced periodontal diseases has been documented during pregnancy, since the late 1800s. The most recent classification of periodontal diseases continues to recognize “pregnancy gingivitis” as a gingival disease induced by biofilm and modified by systemic factors. 2

Women experience hormonal and physiological changes during pregnancy, which have variable effects in the mouth. These changes particularly affect the periodontium in presence or absence of dental plaque/biofilm. During pregnancy, the placenta forms large amounts of estrogen and the human chorionic gonadotrophin that aid in maintaining an increased level of progesterone production (more than 50% higher in pregnant vs. nonpregnant women). In a study by Loee , 121 women in various stages of pregnancy and 61 nonpregnant women showed that severity of gingivitis increased during pregnancy, especially in the second up to eighth month, and then declined to prepregnancy levels. 3 A more recent survey conducted in preconception Chinese women showed 73.9% of participants had periodontal disease; 48% were mild, 51% were moderate, and 1% were severe. 4 Another clinical study also demonstrated that gingivitis and mobility increased during pregnancy and decreased at 3 months postpartum. 5 Furthermore, a longitudinal study monitored salivary proteinase enzyme levels during pregnancy and postpartum. The results showed that bleeding on probing and periodontal pockets were elevated during pregnancy due to a significantly lower level of matrix metalloproteinases-8 concentrations especially during the first and third trimester. But these changes were not statistically significant postpartum. 6 From a microbial standpoint, Kornman and Loesche studied the gingival crevice microbial flora in 20 pregnant and 11 nonpregnant women. The mean gingival index was increased during pregnancy while the plaque index remained unchanged, and *ss. intermedius* increased during pregnancy likely due to steroid hormones that alter the normal subgingival flora. 7 Additionally, a systematic review on the effect of pregnancy on gingival inflammation demonstrated significantly lower gingival index in the first trimester compared with the second and third trimester. 8 Mealey and Moritz inferred that 30 to 100% of pregnant women had pregnancy gingivitis and 10% of these women may develop pyogenic granuloma. Moreover, a selective overgrowth of *Prevotella* *intermedia* due to changes of sex hormones during pregnancy has also been observed. 9

Adverse pregnancy outcomes due to chronic inflammatory conditions of periodontal disease need to be highlighted. Periodontal disease is well documented to be a risk factor for preterm low birth weight (PLBW), low birth weight (LBW), preterm birth (PTB), preeclampsia, decreased birth weight, and shortened gestational age, miscarriage, or stillbirth. 10 Offenbacher et al was the first who found an association between periodontal disease and LBW. 11 In a case–control study to evaluate periodontal disease as a risk factor for PLBW, with 50 volunteer women, it was shown that of all factors examined, only the presence of bacterial vaginosis with periodontitis increased the risk for PLBW by four times. 12 Mothers who had LBW infants were three times more likely to have periodontal pockets ≥ 4 mm and twice as likely to have clinical attachment loss ≥ 3 mm. 13 Likewise, bleeding on probing, probing depth, and clinical attachment loss were more pronounced in mothers who had PTBs when compared to full birth group. 14 Some studies were conducted to evaluate the relationship between gestational diabetes mellitus and periodontal disease. A recent systematic review suggested that periodontal disease is associated with gestational diabetes mellitus, and the risk increased with a family history of diabetes mellitus. 15 The prevalence of gestational diabetes mellitus was 8.3% when compared to the nongestational group, with an increased risk when pregnant women were older, had a significant higher prepregnancy body mass index, or a history of prior gestational diabetes mellitus. 16

In terms of intervention studies, after reviewing 13 randomized clinical trials, 8 studies supported that periodontal treatment had a positive effect on reducing adverse pregnancy outcomes. 17 López et al randomly assigned pregnant women for periodontal treatment before 28 weeks of gestation ( *n* = 200) or to a control group ( *n* = 200) who received periodontal treatment after delivery. The results showed that periodontal treatment significantly reduced the incidence of PLBW babies to about 1.84% compared to the control group which was 10.11%. 18 The result of a retrospective study on the effect of periodontal therapy during pregnancy and the possible adverse pregnancy outcome showed that LBW rate was 5% for women not receiving dental treatment, 4.6% for those who received nonperiodontal and nonprophylactic dental treatment, 3.7% for those who received periodontal treatment before delivery, and 1.8% for those who received postdelivery periodontal treatment. 19 When scaling and root planing, professional prophylaxis and oral hygiene instructions were given to 33 pregnant women during the second trimester, resulting in improvement of periodontal parameters when compared to the nonintervention group. 20 A similar interventional study involving 200 pregnant females were randomly assigned to receive non surgical periodontal therapy, either during gestation, or after completion of delivery. The prevalence of PTB was 53.5% in the treatment group and 76.4% in the control group, while the prevalence of LBW was 26.3% in the treatment group and 53.9% in the control group, concluding that periodontal therapy can reduce the risk of PTB and LWB in mothers affected by periodontitis. 21 Offenbacher et al's pilot study showed significant improvement in all periodontal parameters, lower counts of subgingival plaque bacteria, decrease in gingival crevicular fluid inflammatory mediators, and 3.8-fold reduction in the rate of preterm (PT) delivery in the treatment group when compared to the control group. 22

Many studies have been conducted to evaluate the knowledge of pregnant women about this association and the importance of having regular dental checkups before, during pregnancy, or after delivery. Wu et al designed a questionnaire for pregnant women on factors related to periodontal disease during pregnancy. Note that 74 and 91% pregnant and nonpregnant women, respectively, had little knowledge of an association between pregnancy and oral health, and most of the pregnant women thought it best not to undergo any dental treatment during pregnancy. 23 Moreover, when evaluating the knowledge of an association between pregnancy outcomes and periodontal disease, Tarannum et al showed that younger and more educated pregnant women had more knowledge when compared to older and less educated pregnant women. 24

When general practitioners' knowledge was evaluated in India, results showed that 51% of medical practitioners did not have any information regarding the relationship of periodontal disease with systemic health while in medical college, and 28% have never referred their patients for periodontal evaluation. 25 Gynecologists were surveyed in Riyadh to evaluate the knowledge of periodontal disease and pregnancy outcomes. Fifty-five percent of respondents highlighted that oral hygiene negligence was the main cause of gum disease during pregnancy, while 45% believed that there was a relationship between periodontal disease and PTB. 26 Of 150 private gynecologists who volunteered to participate in a questionnaire survey, 90% had knowledge regarding the association between oral health and pregnancy, and 85% of gynecologists advised their patients to visit a dentist. 27 However, there is scarce evidence present locally regarding knowledge of pregnant females on the association between periodontal health and pregnancy. The aim of this study is to evaluate the knowledge of pregnant females, attending to government-based dental and antenatal clinic based in the United Arab Emirates (UAE), about the relationship between periodontal health/disease and pregnancy and correlation with its sociodemographic data.

## Material and Methods

### Study Design and Criteria for Selection

The survey questionnaire was structured by periodontists (S.A., C.B., and M.S.) at postgraduate periodontology dental practice in English/Arabic. The questions were based on compilation from other previous studies and therefore the questions were validated and piloted. A hard copy printed questionnaire was distributed among pregnant females attending the dental and antenatal clinics in a government setting in the UAE over a period of 7 months from April to October 2017. Consenting pregnant women aged 18 and above, reporting no multiple gestations, and not taking any systemic antibiotics were included in the study. Pregnant females with medical condition such as pregnancy-included hypertension, gestational diabetes, and blood-borne viral infections were excluded from the study.

The questionnaire included 23 items in four sections: six questions on sociodemographic data: age, nationality, educational attainment, occupation, smoking status, and the number of pregnancies. Three questions on oral hygiene habits: tooth-brushing frequency, type, and frequency of dental visit. Four questions on self-reporting oral symptoms during pregnancy: bleeding gums, swelling, halitosis, and loose tooth/teeth; and 10 questions dealt with knowledge concerning periodontal health and its relationship with pregnancy outcomes. A quality of knowledge (QoK) score was developed by giving each “correct” response a value of 1 and each “incorrect” response a value of 0. From question 17 to the last question 23, this was applied except for question 22 which was related to the gynecologist's advice. Therefore, a maximum score of 8 was possible as question 17 had 3 possible “correct” answers. All “correct” responses were summed for each individual and a mean score for each participant and an overall mean score for all respondents was determined. Participants whose individual mean score was below the overall group mean score were categorized as “less knowledge” and those respondents with a mean score above the overall mean were categorized as “more knowledge.”

### Ethical Considerations

Prior to the initiation of the study, ethical approval was obtained from the Institutional Review Board Committee from the Ministry of Health and Prevention research committee for ethical clearance and was registered; the study was conducted in full conformance with ethical principles highlighted by the “Declaration of Helsinki.” 28

### Statistical Analysis

Descriptive statistics were used to provide a clear demonstration of the questionnaire data. Frequency distribution of the participants' oral hygiene habits and distribution of knowledge about periodontal health and its relationship with pregnancy were evaluated. The data was analyzed using chi-square test, analysis of variance and Pearson's correlation coefficient, conducted using Statistical Package for Social Sciences software (SPSS ^##^ , version 24.0, IBM for Mac). The significance level was set at *p* < 0.05.

## Results

A total of 132 participants were recruited for this study. Thirty-two questionnaires were excluded from analysis due to incomplete answers and therefore responses from 100 subjects were analyzed. The average age of the pregnant females was 31.0 years (± 5.9) ranging from 17 to 48 years, and the mean number of pregnancies was 3.3 ± 2.1. Table 1 shows that more than half of the participants were from UAE (64%) and 59% had university level of education, whereas 5% were with primary level of education. The study also showed that the majority (54%) of them were housewives. Table 2 highlights that 65% of the participants brushed their teeth 2 to 3 times per day and the majority used manual toothbrush (93%). Most of them visited the dentist only when in pain (62%), while 6% never visited a dentist. Table 3 represents self-reported oral symptoms during pregnancy. Sixty-two percent reported absence of bleeding gums during pregnancy, and 73% had no swollen gum. The majority of the participants had no bad taste, smell, or odor during pregnancy (66%). Only 21% had lost their tooth/teeth during pregnancy.

**Table 1:** Sociodemographic characteristics of study participants enrolled in the study

Variable		*n* = 100
Q2. Nationality	UAE	64%
	GCC	14%
	Others	22%
Q3. Education	Primary	5%
	Secondary	36%
	University	59%
Q4. Occupation	Housewife	54%
	Student	4%
	Employee	42%
Q5. Smoking	Never	94%
	Ex-smoker	2%
	Current	4%

Abbreviations: GCC, Gulf Cooperation Council; UAE, United Arab Emirates.

**Table 2:** Frequency distribution of the participants' oral hygiene habits

Characteristics		*n* = 100
Q7. How often do you brush your teeth?	Sometimes	6%
	Once a day	29%
	2–3 times per day	65%
Q8. What type of toothbrush do you use?	Manual	93%
	Electric	6%
	Others	1%
Q9. How often do you visit the dentist?	Frequently	22%
	Annually	10%
	In pain	62%
	Never	6%

**Table 3:** Frequency distribution of the participants according to self-reported oral symptoms during pregnancy

Characteristics		*n* = 100
Q10. Do you have bleeding gums?	Yes	38%
	No	62%
Q11. Have your gum become swollen during pregnancy?	Yes	27%
	No	73%
Q12. Do you have a bad taste/smell/odor from the mouth since the pregnancy?	Yes	34%
	No	66%
Q13. Did you have loose tooth/teeth during pregnancy?	Yes	21%
	No	79%

Table 4 displays the knowledge of participants about periodontal health and the relation with pregnancy. More than half of the participants (62%) are aware of the connection between oral health and pregnancy. About 79% believed with the statement “a tooth for a baby.” In addition, 66% believed that a dental checkup was not important during pregnancy. When enquired about the possible cause of inflamed gums during pregnancy, 52% believed that it was due to hormonal changes, 15% due to dental plaque, and 33% due to neglecting tooth brushing; however, most of the response (84%) did not know the cause of inflamed gums. Furthermore, 96% knew the importance of tooth brushing during pregnancy and 53% agreed that pregnancy increases the likelihood of gum disease while 15% of participants believed that gum disease will not lead to PT or low weight (LW) infants. The majority (94%) knew that smoking has a negative effect on the pregnant female and the baby. This study also showed that the majority of gynecologists (66%) do not advise their pregnant patients to visit the dentist during pregnancy. Most of them (31%) regarded the second trimester as the safest trimester to receive dental treatment.

**Table 4:** Frequency distribution of knowledge about periodontal health and the relation with pregnancy

Question			*n* = 100
Q14. Have you heard about the connection between oral health and pregnancy?		Yes	62%
		No	38%
Q15. Do you believe in the statement “a tooth for a baby”?		Yes	79%
		No	31%
Q16. Do you think a dental checkup is important during pregnancy?		Yes	34%
		No	66%
Q17. Which of the following do you think may cause inflamed gums during pregnancy?	Hormonal changes	Yes	52%
		No	48%
	Dental plaque	Yes	15%
		No	85%
	Neglecting tooth brushing	Yes	33%
		No	67%
	Don't know	Yes	16%
		No	84%
Q18. Do you think tooth brushing is important during pregnancy?		Yes	96%
		No	4%
Q19. Do you think pregnancy increases the likelihood of gum disease?		Yes	53%
		No	47%
Q20. Do you think gum disease leads to a preterm or low-weight infant?		Yes	15%
		No	85%
Q21. Do you think smoking has a negative effect on the pregnant mother and baby?		Yes	94%
		No	6%
Q22. Does your gynecologist advise you to visit dentist during pregnancy?		Yes	34%
		No	66%
Q23. Which trimester do you think is the safest trimester for dental treatment?		First trimester	21%
		Second trimester	31%
		Third trimester	22%
		Never visit a dentist during pregnancy	26%

Table 5 represents the frequency distribution of the QoK with demographic data. It was observed that nationality and educational attainment had no significant association with the QoK of pregnant women. Housewives were significantly less knowledgeable about oral health compared to students and/or employed pregnant females ( *p* = 0.011). Interpretation of the results for smokers must be treated with caution because of the low number of ex-smoker/smoker pregnant females. Older participants (age more than 30 years) were more likely to believe in “a tooth for a baby” than younger participants ( *p* < 0.05) ( Table 6a ). The relationship between age and education with this belief was analyzed by Fisher's exact test, and no significant difference was found, *p* = 0.363 ( Table 6b ). However, 70 out of 100 respondents believed in “a tooth for a baby.” The model shows that educational attainment was not a predictor for the belief “a tooth for a baby,” but age was a significant predictor, odds ratio = 2.0 (95% confidence interval = 1.24, 3.2), *p* < 0.01 ( Table 6c ).

**Table 5:** Frequency distributions of the quality of knowledge with demographic data

Variable		Quality of knowledge (QoK)		*p* -Value
		Less knowledgeable	More knowledgeable	
Nationality	UAE	39 (60.9%)	25 (39.1%)	0.217
	GCC	7 (50.0%)	7 (50.0%)	
	Others	17 (77.3%)	5 (22.7%)	
Education	School only	30 (73.2%)	11 (26.8%)	0.060
	University and plus	33 (55.9%)	26 (44.1%)	
Occupation	Housewife	40 (74.1%)	14 (25.9%)	0.011 a
	Student/ employee	23 (50.0%)	23 (50.0%)	
Smoking	Nonsmoker	62 (66.0%)	32 (34.0%)	0.025 a
	Ex-smoker/smoker	1 (16.7%)	5 (83.8%)	

Abbreviations: GCC, Gulf Cooperation Council; UAE, United Arab Emirates.

a *p* < 0.05.

**Table 6:** (a) Age and belief in “a tooth for a baby”; (b) educational attainment and the belief in “a tooth for a baby”; and (c) logistic regression with “a tooth for a baby” as the dependent variable and age and education as the predictor variables

	Age in years	A tooth for a baby		Chi-square	
**(a)**		**No**	**Yes**		
	≤ 30	21	29	9.5, *p* < 0.05 a	
	> 30	9	41		
**(b)**	**Education**	**A tooth for a baby**		**Total**	
		**No**	**Yes**		
	School only	11	30	41	
	University	19	40	59	
	Total	30	70	100	
**(c)**		**Logistic regression coefficient B**	**Standard error**	**Significance**	**Odds ratio**
	Education	–0.19	0.472	0.687	0.83
	Age	0.689	0.244	0.005 a	2

a *p* <0.05.

## Discussion

Hormonal changes during pregnancy have a significant impact on oral health, especially if oral health is neglected. Therefore, pregnant females should be aware of the relationship between pregnancy and periodontal health. This study assessed the knowledge of pregnant women about periodontal disease during pregnancy. There was no association between educational attainment and “score of knowledge” for pregnant females ( *p* = 0.06). A cross-sectional study by Alwaeli and Al-Jundi in 2005 also showed that having good knowledge about periodontal health was unrelated to education level. 29 Moreover, Taani et al evaluated the clinical status of the periodontium in 400 pregnant and nonpregnant females and found that the lower educational attainment was not associated with greater probing depth. 30

The majority of participants in this study were housewives (54%), followed by employed participants (42%) and students (4%). Housewives were significantly less knowledgeable than students and employed pregnant females ( *p* = 0.011). This is in contrast to the findings by Ghazal et al who found no significant association between gingival heath and occupation in a group of housewives. 31 Contrarily, Navkiran et al, Dhaliwal et al, and Machuca et al identified a significant relationship between periodontal parameters and sociodemographic status. 32 33 34

Smoking during pregnancy and its deleterious effects on the mother and the unborn such as LBW, PT delivery, and respiratory infections have been documented. 35 Wang et al showed that mothers who continuously smoked during pregnancy had increased instance of LBW infants compared with infants of nonsmokers. 36 37 The current study demonstrated a minor 4% of recruits as current smokers. In terms of oral home care regimen, 65% of pregnant females brushed their teeth 2 to 3 times daily, similar to a study by Hashim who reported 66.5% brushed their teeth 2 to 3 times daily, while 29% brushed once a day compared to 27.6% in our study. 38 In addition, 93% of pregnant females used a manual toothbrush. Systematic reviews have deliberated that a powered toothbrush was at least as effective as a manual toothbrush in terms of reducing dental plaque and that a single session of oral home care was effective in improving mechanical plaque removal by a manual toothbrush. 39 40 Moreover, a recent cross-sectional review have confirmed an overall lack of application of adjunctive tooth brushing practices like interdental aids and mouthwashes in pregnant women within UAE. 41 While a majority of participants (65%) demonstrated satisfactory oral home care routine, there is a need to emphasize the importance of introducing interdental brushes for mechanical plaque control, that aid in disruption of biofilm from the interdental spaces, accounting for around 30% of all teeth surfaces.

More than half of pregnant females visited a dentist only with pain prior, during, or after pregnancy, while 6% had never visited a dentist. A cross-sectional study of pregnant females in Yazd, Iran, showed that 59% visited a dentist due to dental pain, 18% for a dental checkup, and 4% due to gingival bleeding. 42 Another cross-sectional study conducted in Kuwait showed that 52% of participants visited the dentist during pregnancy and less than half (30%) due to pain. 43 Additionally, only 18% of pregnant females who visited the Ministry of Health hospitals in Dammam visited the dentist before or after pregnancy. 44 A study by Al Habashnehet al assessed factors related to utilization of dental services during pregnancy. Older mothers, high levels of education, healthier lifestyle behaviors, those who used dental aids for oral hygiene practice, and nonsmoker mothers were more likely to have visited a dentist during pregnancy. 45 Another study assessed the reason for not utilizing dental services during pregnancy. Two barriers were identified: stress (emotional, physical, and financial) and dental-related stress (previous experience, pain, long waiting time). 46 In the present study, pregnant females practiced daily oral hygiene routine and understood the importance of daily tooth brushing, but most of them did not have sufficient knowledge regarding the importance of an annual dental checkup and sought dental treatment only when symptoms appeared.

Less than half of participants in our study reported gingival and dental symptoms during pregnancy. This is consistent with the findings of Hashim who showed 24% of participants reported having “gum problems.” 38 When assessing oral health status among pregnant females, 96% reported bleeding gums, 33% burning gums, 25% swollen gums, and 31% loose teeth. 47 George et al identified 78% of pregnant females reporting bleeding gums, 22% toothache, and 4% loose teeth. 48 An oral examination of 1,224 pregnant women showed a significant increase in clinical attachment loss and pocket depth indicating some form of periodontal disease progression during pregnancy; additionally, when comparing oral conditions between pregnant and nonpregnant females, pregnant women showed a higher incidence of dental caries, gingivitis, and pyogenic granuloma. 49 50 Periodontal status improved after delivery as observed by González-Jaranay et al, plaque index, gingival index, and probing depth significantly improved between baseline and postpartum. 51

In the present study, most participants maintained their daily oral home care practice and did not report gingival symptoms during pregnancy despite the fact that they did not visit a dental clinic regularly. In the present survey, 62% of pregnant females knew the connection between oral health and periodontal disease—similar to other studies, where the knowledge between pregnancy and oral health ranged between 60 and 75%. 38 52 In contrast, studies done in Saudi Arabia and Jordan showed that pregnant participants had little or limited knowledge about periodontal disease during pregnancy. 29 53 However, 66% of pregnant females in the present study did not believe that a regular dental checkup was important during pregnancy. This might be attributed to the fact that most people in the survey do not attend regular dental checkups. As a result, tooth extraction during pregnancy was common and had given rise to the belief of “a tooth for a baby.” Anecdotal opinion also suggested that pregnant women also believe that “babies in the womb absorb maternal calcium from their bones and teeth.” While the study found that the majority of pregnant women surveyed in government-based settings in the UAE had good knowledge about periodontal health and pregnancy, there is still a significant portion of the population, around 40%, who remain unaware of this correlation. It is imperative to provide education to this group on the importance of periodontal health during pregnancy.

Several factors can increase inflammation of gums during pregnancy: hormonal changes, dental plaque, and neglecting tooth brushing. In our study, half of the participants agreed that hormonal changes cause inflamed gums while 15% knew that dental plaque causes inflamed gums. This is contrary to Asa'ad et al and Alwaeli and Al-Jundi who found that 30 and 22.5%, respectively, were aware that dental plaque causes inflamed gums. 29 53 More than half of the present participants (67%) did not believe that neglecting tooth brushing caused inflamed gums, less than 39%, reported by Asa'ad et al. 53

Neglecting oral health during pregnancy has also been associated with birth complications such as PT or LW infants. 27 However, patient perception regarding this correlation between poor oral home care and its effect on PT or LW births is questionable. Our study demonstrated that only 15% of pregnant women believed that gingival inflammation may lead to delivery of PT or LW infants. This low percentage of patient knowledge is in accordance to findings from other populations within the region (5–11%). 29 41 53 Most of the participants in the present study were aware that smoking has a negative effect on the pregnant mother and baby, which agreed with other findings. 29 53

With regards to the contribution of health care professionals toward guiding pregnant patients to achieve oral health stability, most gynecologists (82%) in a cross-sectional study advised their pregnant patients to go for a dental checkup. 27 This is in contrast to our findings, where surveyed participants did not recollect their gynecologists' advice regarding dental visits; 66% stated that visit to a dental clinic was not advised. Another survey based in North Carolina showed that only 51% of obstetricians recommended a dental checkup during pregnancy. 54 Surprisingly, medical physicians in Jordan, when asked about their knowledge of association between oral health and pregnancy outcomes, majority (88%) advised their pregnant females to delay their dental treatment and about half of them did not advise a dental checkup during pregnancy. 55 On the topic of timing of dental visits, results from Hashim and Akbar showed that the majority of gynecologists considered the second trimester to be the safest trimester for dental treatment. 27 In contrast, only 31% of respondents in this study believed that the second trimester is the safest trimester. Pakistani researchers aimed to assess the knowledge of dentists with different educational qualifications related to dental treatment for pregnant females and showed that general dentists tended to not treat pregnant females while postgraduate residents would treat them. Also, clinicians with less than 3 years' experience believed that dental treatment can be done at any trimester. 56 Moreover, studies have shown that the second trimester is the safest trimester for dental treatment. 57 58 These inconsistencies in knowledge and awareness among medical professionals, underlines the need to incorporate interprofessional collaborative practice as an essential component in effective prevention, early detection, and delivering optimum care for our female patients. The approach to integrate oral health as part of overall health care education and design a curriculum that blends dental awareness with patient's overall well-being is paramount. Following that, numerous international health care organizations like the American Academy of Periodontology, the American College of Obstetricians and Gynecologists, the American College of Nurse-Midwives, and the American Dental Association have issued consensus reports and recommendations for improving oral health care during pregnancy. These guidelines can aid health care professionals across the world to collaborate with their oral health counterparts to provide comprehensive health care to their women patients. Limitations of this survey study may be attributed to a small sample size, that precludes extrapolating the results to all pregnant females in the region. Furthermore, the findings may not be generalized, as the subjects were recruited from a limited cohort, who attended the government settings during a restricted timeline.

## Conclusion

Our results showed that the majority of pregnant females surveyed in government-based settings in the UAE had good knowledge about periodontal health and pregnancy. Pregnant housewives tended to have less knowledge about periodontal disease and pregnancy than employed and student respondents. Age was a predictor for the belief in “a tooth for a baby,” mainly in respondents > 30 years of age. More than half of the respondents did not believe that gum diseases were associated with PT or LW infants. Most of the gynecologists did not advise pregnant patients to have a dental checkup during pregnancy. It is recommended that enhanced collaborations between antenatal clinics and preventive dental settings can provide a holistic approach toward improving overall health for pregnant females.
